# Curriculum factors influencing knowledge of communication skills among medical students

**DOI:** 10.1186/1472-6920-7-35

**Published:** 2007-10-10

**Authors:** Anders Baerheim, Per Hjortdahl, Are Holen, Tor Anvik, Ole Bernt Fasmer, Hilde Grimstad, Tore Gude, Terje Risberg, Per Vaglum

**Affiliations:** 1 Department of Public Health and Primary Health Care, University of Bergen, Norway; 2 Institute of Clinical Medicine, University of Bergen, Norway; 3 Institute of General Practice and Community Medicine University of Oslo, Norway; 4 Department of Behavioural Sciences in Medicine, University of Oslo, Norway; 5 Department of Public Health and General Practice, University of Science and Technology, Trondheim, Norway; 6 Department of Neuroscience, University of Science and Technology, Trondheim, Norway; 7 Department of Community Medicine, University of Tromsø, Norway, Trondheim, Norway; 8 Department of Oncology, University of Tromsø, Norway, Trondheim, Norway

## Abstract

**Background:**

Communication training builds on the assumption that understanding of the concepts related to professional communication facilitates the training. We know little about whether students' knowledge of clinical communication skills is affected by their attendance of communication training courses, or to what degree other elements of the clinical training or curriculum design also play a role. The aim of this study was to determine which elements of the curriculum influence acquisition of knowledge regarding clinical communication skills by medical students.

**Methods:**

The study design was a cross-sectional survey performed in the four Norwegian medical schools with different curricula, spring 2003. A self-administered questionnaire regarding knowledge of communication skills (an abridged version of van Dalen's paper-and-pencil test) was sent to all students attending the four medical schools. A total of 1801 (59%) students responded with complete questionnaires.

**Results:**

At the end of the 1st year of study, the score on the knowledge test was higher in students at the two schools running communication courses and providing early patient contact (mean 81%) than in the other two medical schools (mean 69–75%, *P *≤ 0.001), with students studying a traditional curriculum scoring the lowest. Their scores increased sharply towards the end of the 3rd year, during which they had been subjected to extensive patient contact and had participated in an intensive communication course (77% vs. 72% the previous year, *P *≤ 0.01). All students scored generally lower in academic years in which there was no communication training. However, at the end of the final year the difference between the schools was only 5% (81% vs. 86%, *P *≤ 0.001).

**Conclusion:**

The acquisition of knowledge regarding communication skills by medical students may be optimised when the training is given together with extensive supervised patient contact, especially if this teaching takes place in the initial years of the curriculum.

## Background

Excellent communication skills are essential to medical professionalism [1]. Clinical communication is complex in nature, and both personal and curricular factors will influence how medical students master the relevant skills [2]. Basic or general communication skills are developed early in life. Theoretical knowledge about communication skills comes years later, and not through medical studies alone. Contrary to clinical procedural skills, communication skills appear to be an integral part of one's cognition [3].

Communication training builds on the assumption that understanding of the concepts related to professional communication facilitates the training [4,5]. Although knowledge alone is insufficient for actual behaviour change [6], theoretical knowledge may help the students to achieve their training goals.

Van Dalen *et al*. constructed the 'paper-and-pencil test' used to explore students' knowledge about clinical communication skills [7]. While they have found the test to be a predictor of performance of such skills, the correlation between 'knowing how' and 'showing' is weaker than what has been shown for clinical procedural skills [7-9].

We know little about whether students' knowledge of clinical communication skills is affected by their attendance of communication training courses, or to what degree other elements of the clinical training or curriculum design also play a role.

The aim of this nationwide, cross-sectional study was to determine which curriculum elements influence the acquisition of knowledge regarding clinical communication skills by medical students.

## Methods

### Participants

In spring 2003, all medical students (*N *= 3055) at the four Norwegian medical schools (in all six annual classes) were invited to participate in a cross-sectional postal survey. Questionnaires were distributed in May and June, at the end of the academic year. After two reminders (one by letter and one by e-mail), 1833 (60%) of the students responded. Of these, 32 students had not completed central parameters. Accordingly, 1801 self-completed questionnaires were investigated (Table 1).

**Table 1:** Response rates as percentages among 3055 students in the four Norwegian medical schools as a function of academic year

**School/year**	**Early/PBL A**		**Early/PBL B**		**Integrated**		**Traditional**	
1	60	(60)	61	(64)	50	(60)	45	(45)
2	54	(50)	64	(79)	48	(62)	54	(59)
3	55	(60)	56	(56)	54	(57)	68	(73)
4	47	(53)	62	(68)	71	(73)	54	(58)
5	54	(72)	60	(79)	55	(61)	65	(66)
6	49	(59)	65	(71)	56	(71)	62	(68)

Students (N)	566		1108		496		885	
Responders (N)	308		689		279		525	
Mean response rate	54%		62%		56%		59%	

Of the respondents, 1801 had completed the necessary parameters on the questionnaire. Responses for female students are given as percentages in parentheses.

#### Differences in curricula

The four medical schools follow different curricula. Two employ problem-based learning (PBL) and involve early patient contact. These two schools are denoted 'Early/PBL A' and 'Early/PBL B'; both run a doctor-patient course during the 1st year of study (the first 2 years for school A). In this course, students are allocated to family doctors, with training twice monthly to learn how to interview patients about their background, medical history, expectations regarding the consultation, understanding of their symptoms and their emotions.

The third school follows an integrated curriculum with parallel preclinical and clinical training, but does not use PBL and does not run an extensive doctor-patient course during the 1st years of study. This school is denoted as 'Integrated'.

The fourth school is traditional, with a sharp division between preclinical and clinical training after 2.25 years. This school is denoted as 'Traditional'. The two PBL-based schools and the Integrated school provide clinical teaching during all 6 years of study, while students at the Traditional school experience their first patient contact at the beginning of the 3rd year.

All schools have a common, national admittance system. About 10% of applicants are admitted annually. The criteria for admittance are identical in the four schools. Students are selected based on study points from previous studies, range 58.9–61.8 points in 2003 [10]. All schools provide a 6-year curriculum.

Intensified clinical training in primary care preceptorship and hospital-based preceptorship (mainly in smaller hospitals) takes place in the 5th year in the Integrated school (8 weeks in a primary-care setting vs. 16 weeks in a hospital) and in school Early/PBL B (6 weeks vs. 6 weeks), and in the 5th and 6th years in school Early/PBL A (6 weeks vs. 16 weeks). Similar teaching is spread through years 4–6 at the Traditional school (4 weeks vs. 15 weeks) [11]. The master-apprentice approach is the main didactic element in preceptorship-based training, whether it takes place in primary care or in smaller hospitals. Students learn by observing, performing and reflecting, all as a part of clinical work. In primary care, each student is allocated to one general practitioner, and in the smaller hospitals, limited groups of students (5–10 students per group) enter the different departments.

The timing and volume of communication training elements in the curricula at the four medical schools are given in Table 2. In all communication training sessions, students are given observation-based feedback. Most (50–70%) of the students usually participate in non-compulsory courses (Table 2).

**Table 2:** Placement and duration (in hours) of communication training in the curricula of the four Norwegian medical schools

School	Early/PBL A	Early/PBL B	Integrated	Traditional
Year 1	**24**^z^	**7**^z^	4	0
Year 2	**16**^z^	**7**^y^	0	0
Year 3	0	**25**^y^	**14**	**24**^x, y^
Year 4	0	0	2	0^x^
Year 5	0^x^	0^x^	0^x^	24^x^
Year 6	**5**^x, y^	18^y^	12	**16**^x, y^

Totals	45	57	32	64

Mandatory courses are given in *bold*. Courses based on encounters with real or simulated patients are marked, as are periods with supervised patient contact during preceptorship or a doctor-patient course (see text for details).

^x^Years with supervised patient contact in preceptorship

^y^Use of simulated patients or real patients during communication course

^z^Doctor-patient course, 6 h/month; 2 years at Early/PBL A school; 1 year at Early/PBL B school.

#### Instrument

The main instrument used in this study is based on the paper-and-pencil test as described by van Dalen *et al *[7]. Originally, this questionnaire comprised 78 items addressing theoretical and conceptual knowledge regarding communication skills. The aim of the questionnaire is to establish whether students can identify the communication interventions that are most effective in certain situations [7]. Items are formed as either short, context-based questions, or they are in the format of a short vignette followed by one or more statements. Students mark each statement as true or false. The sum of the test score is presented as a percentage [7].

Examples of questions are:

Imagine that in a consultation with a patient with lung cancer you have advised him to stop smoking. He responds by saying: 'I will try. But you think it's easy, don't you?'. The doctor could respond in several different ways:

28. It is better not to ask at all than to say 'Do you think you can do it?'; *true/false*

29. To ask 'How do you plan to do it?' is better than saying 'It will be hard to stop'; *true/false*.

The questionnaire was translated from Dutch to Norwegian by a bilingual Dutch psychologist and a bilingual Norwegian medical communication trainer. The questionnaire was tested on ten medical communication teachers. Of a total of 78 items, 34 were selected because they produced the most consistent scoring. On further analysis of the abridged questionnaire, four more items were deleted because they were deemed not relevant to our study. The final result was a 30-item questionnaire covering communicative microskills, clinical interaction, patient-centred style, the provision of information and the breaking of bad news.

Data on the students' exposure to communication training or preceptorship in general practice or local hospitals were extracted from the curricula of the four medical schools. Background variables comprised gender, previous health-related education, and work experiences in the health services undertaken prior to medical school (yes/no).

Ethical approval was not deemed necessary.

### Statistical analysis

The mean scores for knowledge of communication skills were calculated and converted to percentages [7]. Differences between groups were tested with independent *t*-test or with one-factor or two-factor ANOVA. A significance level of 0.05 was chosen. The SPSS statistical package (version 11.0) was used in the analyses.

## Results

The mean age of the 1801 respondents was 24.7 years (range 19–48 years). Sixty-four percent of the students were female (range among the four schools 61–66%), 12% (range 11–17%) had completed another health-training program (e.g. nursing or physiotherapy) and 42% (range 38–45%) had worked as health workers prior to attending medical school. These background variables did not differ significantly between the students at the four schools or between the academic years. During their medical studies, 73% (range 70–80%) of the students had undertaken health-related extra-curricular work.

Overall, the student scores for knowledge of communication skills varied substantially depending upon which school they attended and which academic year they were in (Table 3, *P *≤ 0.001, two-factor ANOVA). The effect of gender was small, but statistically significant, females scoring 1.7% higher than males (79.9% vs. 78.2%, *P *≤ 0.001, *t*-test).

**Table 3:** Medical students' mean scores on a 30-item test of knowledge of communication skills

School:	Early/PBL A	Early/PBL B	Integrated	Traditional
Year 1	81.2	81.0	74.5	69.3
Year 2	82.4	78.8	76.0	71.8
Year 3	82.7	80.9	79.2	76.6**
Year 4	79.3	79.3	80.3	77.0
Year 5	83.7*	81.7*	82.1	78.2
Year 6	85.7	83.4	81.0	80.5

Increase from years 1 to 6	4.5	2.4	6.5^§^	11.2^§^

Two schools provide early patient contact and a PBL-based curriculum (Early/PBL A and Early/PBL B), one school follows an integrated curriculum (Integrated) and the fourth follows a traditional curriculum (Traditional). Differences between schools were significant for all 6 years (*P *≤ 0.001).

* Schools Early/PBL A and Early/PBL B, year 5 vs. year 4, *P *≤ 0.05, independent *t*-test

**Traditional school, year 3 vs. year 2; *P *≤ 0.01, independent *t*-test

§*P *≤ 0.001, ANOVA

The effect of the curriculum type was observed most clearly by the end of the 1st year, when the scores for knowledge of communication skills was much high for students at Early/PBL A and Early/PBL B schools than for students at the other two schools (Table 3, 81% vs. 75% and 69%, *P*= 0.001, ANOVA). The Early/PBL schools give 1st-year students extensive early patient contact combined with communication training.

The Early/PBL A and Early/PBL B schools provide no communication training in the 4th year (Table 2). At the end of that year, scores at these two schools (both 79%) were lower than they had been at the end of the 3rd year (83%, 80%) and were significantly lower than that at the end of the 5th year (84%, 82%; *P *≤ 0.05, *t*-test). In the 5th year, both schools offer extensive preceptorship, but still no specific communication training. At the end of year 6, students of these two schools did not score any higher than at the end of year 1 (Table 3).

Students at the Integrated school receive limited communication training and patient contact during the 1st year, and they scored at an intermediate level at the end of this year. Scores increased gradually over the subsequent years, with no statistically significant leaps (years 1–6, *P *≤ 0.001).

Students at the Traditional school scored the lowest among the schools at the end of the 1st year (Table 3). This school follows a traditional curriculum with no patient contact or communication training the first 2 years. However, scores increased sharply in the 3rd year, when they had their first and extensive patient contact and a 24-h communication course (77% vs. 72% the previous year, *P *≤ 0.01, *t*-test). Scores at this school also increased significantly from year 4 to year 6 (*P *≤ 0.05, ANOVA), and reached the level of the Integrated school during the 6th year. During years 4–6, students at the Traditional school are subjected to far more communication training than the other students and have some clinical preceptorship each year (Table 2).

The difference between the four schools, although still statistically significant (*P *≤ 0.001, ANOVA), was far less at the end of year 6 than at the end of year 1 (5% vs. 12%). Schools with the lowest scores at the end of the 1st year showed the highest increase towards the 6th year, but did not become level with those of the two Early/PBL schools.

## Discussion

Our results indicate that communication training combined with supervised clinical work with patients is most strongly correlated with high scores for knowledge of clinical communication skills, especially when the combination takes place early in the medical school curriculum.

### Strengths and limitations of the study

The present study employed a nationwide cross-sectional survey involving all four medical schools in Norway. Subjects were present at all stages of training, which allowed subanalyses of the results at the level of the relevant curriculum elements.

Several factors may limit the validity of our findings. A higher response rate might have resulted in different scores, since scores often differ between responders and non-responders [12]. Also, non-responders may be academically weaker [13].

Our questionnaire is an abridged version of the test of van Dalen *et al*., and the two questionnaires exhibit similar internal consistency [7]. However, the scores of the students at our medical schools were substantially higher (at year 6; 81–86%) than those of students at the same level in Maastricht and Leiden, The Netherlands (52–59%) [7]. This may be attributable to the selection of items that obtained the most consistent scoring in our pilot study. Consequently, our results cannot be compared with those of van Dalen *et al*. Nevertheless, the same questionnaire was applied throughout our study, and the differences in scores related to the effects of curriculum elements should be valid.

Our data are not suitable for analysing the potential importance of supervised clinical work in preceptorship or communication courses alone. Linear regression proved futile, since the *R*^2 ^value was 0.02, probably because the entered curriculum variables were strongly clustered in accordance with the curriculum. However, the effect of the combination of preceptorship and communication training is striking, especially if it takes place early in the study (Early/PBL schools) or early in the clinical part of the study (Traditional school).

There is also a close relationship between 1st-year early patient contact, communication training and the PBL-based curriculum, as only the two Early/PBL schools have these curricular elements. We therefore cannot rule out any crossover effect of being exposed to PBL-based training and an increased knowledge of communication skills.

#### Discussion of results

The four medical schools in Norway – one Traditional, one Integrated and two based on PBL – may together represent the contemporary varieties of medical schools in the Western world. Due to the common admittance system in Norway, 1st-year students at the four schools are comparable at the time of admittance. The similarities between the students' background variables reinforce this view.

Early communication training and extensive patient contact during the 1st year of medical school increased the scores on knowledge of communication skills to an extent unparalleled later in the curricula by any of the four schools, even though the quantity of later training in the same curricular elements is far greater. These results should be interpreted with some caution, since for the Early/PBL schools, PBL pedagogy may in itself account for some of our findings; PBL activity may be a confounder.

Early communication training has been advocated by, among others, Deveugele *et al *[14]. Fineberg reports that early intervention increases the proficiency and skills needed to conduct family conferences and advances communication in palliative care [15]. For acquisition of knowledge of communication skills, our results indicate that early extensive supervised patient contact combined with smaller amounts of communication training is more effective than more extensive communication training later in the curriculum.

Maguire and Pitceathly have shown that successful training in communication skills depends partly on the training itself and partly on its contextual relevance (i.e. adequate patient exposure) [16]. Our results indicate that knowledge of communication skills is best attained by attendance of communication courses in combination with clinical training. First-year students at the Early/PBL schools may be an example of this. Another example is the substantial increase in scores from the end of the 2nd year to the end of the 3rd year in the Traditional school. Students at this school start their clinical training during the 3rd year of study, which is also when they have their first communication course.

Furthermore, at the two Early/PBL schools, there is no communication training in year 4, although clinical teaching involving patients is maintained. Students from these schools scored lower at the end of year 4 compared to year 3, and also to year 5, in which they are exposed to extensive preceptorship. One interpretation may be that knowledge of communication skills may be transitory in nature. However, as the 5th year preceptorship focuses on practical supervised training with few theoretical elements and little or no formal communication training, it would appear likely that knowledge of communication skills is more easily recalled when students work in a clinical context with extensive patient contact.

There is a 5% difference in scores at end of the 6th year. The scores of students at the Traditional and the Integrated schools increased significantly, while there was less change in the scores of those from the two Early/PBL schools. The difference is highly statistically significant, but is it also relevant? One-twentieth of the total score range may appear unimpressive as an effect difference; however, it is not an uncommon finding when evaluating the effects of feedback-based communication training [17].

Improved knowledge and attitudes are not sufficient in themselves to change behaviour in daily practice; practical training is also needed [18]. However, just as theoretical knowledge about practical procedural skills is needed in training, relevant theoretical knowledge may also help students when training communication skills. Hulsman *et al*. reported that the success of communication skills training depends on whether the trainees intend to change their behaviour [6]. An intention to change may be more easily formed when the trainee has distinct concepts regarding what to train. Consequently, we consider it likely that knowledge of communication skills will increase the success of training such skills, although this notion requires verification by further research.

## Conclusion

The results from this study suggest that acquisition of knowledge related to communication skills by medical students will be optimised when the training is given together with extensive supervised patient contact, especially if this teaching takes place in the initial years of the medical school curriculum.

## Competing interests

The author(s) declare that they have no competing interests.

## Authors' contributions

All authors participated in the design of the study and data collection. AB performed the statistical analyses and drafted the manuscript. All authors read and approved the final manuscript.

## Pre-publication history

The pre-publication history for this paper can be accessed here:



## References

[B1] Stern DT (2006). Measuring medical professionalism.

[B2] Noble L (2002). Communication skills training: pragmatism versus proof. Med Educ.

[B3] Lakoff G, Johnson M (1999). Philosophy in the flesh.

[B4] Kurtz SM (2002). Doctor-patient communication: principles and practices. Can J Neurol Sci.

[B5] Howells RJ, Davis HA, Silverman JD (2006). Teaching and learning consultation skills for paediatric practice. Arch Dis Child.

[B6] Hulsman RL, Ros WJ, Winnubst JA, Bensing JM (2002). The effectiveness of a computer-assisted instruction programme on communication skills of medical specialists in oncology. Med Educ.

[B7] Van Dalen J, Kerkhofs E, Verwijnen GM, van Knippenberg-van den Berg BW, van den Hout HA, Scherpbier AJ, van der Vleuten CP (2002). Predicting communication skills with a paper-and-pencil test. Med Educ.

[B8] Janssen JJ, Tan LH, van der Vleuten CP, van Luijk SD, Rethans JJ, Grol RP (1995). Assessment of competence in technical clinical skills of general practitioners. Med Educ.

[B9] Remmen R, Scherpbier A, Denekens J, Derese A, Hermann I, Hoogenboom R, van der Vleuten C, van Royen P, Bossaert L (2001). Correlation of a written test of skills and a performance based test: a study in two traditional medical schools. Med Teach.

[B10] Opptaksgrenser Opptakssentralen for medisinstudiene i Bergen, Oslo, Tromsø og Trondheim. http://www.ntnu.no/studieavd/medisin/opptaksgrenser.html.

[B11] Gude T, Anvik T, Baerheim A, Fasmer OB, Grimstad H, Hjortdahl P, Holen A, Risberg T, Vaglum P (2003). Teaching clinical communication to medical students in Norway (English abstract). Tidsskr Nor Laegeforen.

[B12] Traugott MW (1987). The importance of persistence in respondent selection for pre-election surveys. Public Opin Quart.

[B13] Rudland JR, Pippard MJ, Rennie SC (2005). Comparison of opinions and profiles of late and non-responding medical students with initial responders to a course evaluation questionnaire. Med Teach.

[B14] Deveugele M, Derese A, De Maesschalck S, Willems S, Van Driel M, De Maeseneer J (2005). Teaching communication skills to medical students, a challenge in the curriculum?. Patient Educ Couns.

[B15] Fineberg IC (2005). Preparing professionals for family conferences in palliative care: evaluation results of an interdisciplinary approach. J Palliat Med.

[B16] Maguire P, Pitceathly C (2002). Key communication skills and how to acquire them. BMJ.

[B17] Yedidia MJ, Gillespie CC, Kachur E, Schwartz MD, Ockene J, Chepaitis AE, Snyder CW, Lazare A, Lipkin M (2003). Effect of communications training on medical student performance. JAMA.

[B18] Francke AL, Garssen B, Huijer Abu-Saad H (1995). Determinants of change in nurses' behaviour after continuing education: a literature review. J Adv Nurs.

